# Transcriptome-wide mining suggests conglomerate of genes associated with tuberous root growth and development in *Aconitum heterophyllum* Wall

**DOI:** 10.1007/s13205-016-0466-y

**Published:** 2016-07-11

**Authors:** Nikhil Malhotra, Hemant Sood, Rajinder Singh Chauhan

**Affiliations:** Department of Biotechnology and Bioinformatics, Jaypee University of Information Technology, Waknaghat, Himachal Pradesh 173234 India

**Keywords:** *Aconitum heterophyllum*, Biomass, Primary metabolites, Transcriptome, Transcript abundance, Tuberous root

## Abstract

Tuberous roots of *Aconitum heterophyllum* constitute storage organ for secondary metabolites, however, molecular components contributing to their formation are not known. The transcriptomes of *A. heterophyllum* were analyzed to identify possible genes associated with tuberous root development by taking clues from genes implicated in other plant species. Out of 18 genes, eight genes encoding GDP-mannose pyrophosphorylase (GMPase), SHAGGY, Expansin, RING-box protein 1 (RBX1), SRF receptor kinase (SRF), β-amylase, ADP-glucose pyrophosphorylase (AGPase) and Auxin responsive factor 2 (ARF2) showed higher transcript abundance in roots (13–171 folds) compared to shoots. Comparative expression analysis of those genes between tuberous root developmental stages showed 11–97 folds increase in transcripts in fully developed roots compared to young rootlets, thereby implying their association in biosynthesis, accumulation and storage of primary metabolites towards root biomass. Cluster analysis revealed a positive correlation with the gene expression data for different stages of tuberous root formation in *A. heterophyllum*. The outcome of this study can be useful in genetic improvement of *A. heterophyllum* for root biomass yield.

**Electronic supplementary material:**

The online version of this article (doi:10.1007/s13205-016-0466-y) contains supplementary material, which is available to authorized users.

## Introduction

*Aconitum heterophyllum* Wall ex Royle (Ranunculaceae), popularly known as Atis, is a biennial herb native to North-Western and Eastern Himalayas of India. Its tuberous roots are commonly used as therapeutic ingredient in the traditional indian medicinal system for curing dyspepsia, abdominal pain, diabetes and diarrhea (Kumar et al. 2016). Non-toxic active components like atisine, hetisine and heteratisine (Pelletier et al. 1968; Malhotra et al. 2014) accumulating in tuberous roots of *A. heterophyllum* have wide pharmacological effects on immune, digestive and nervous systems (Rastogi and Mehrotra 1991; Sojitra et al. 2013).

Tuberization in *A. heterophyllum* is a distinctive process from young rootlet to fully mature storage roots which are committed to the storage of primary as well as secondary metabolites. Several studies on morphogenesis have been carried out to understand tuberous root development in various plant species such as *Ipomoea batatas* and *Manihot esculenta* (Indira and Kurian 1977; Wang et al. 2005). Multiple genes, such as MADS-box, NAM-like and SRF have been shown to regulate tuberous root development in these plant species (You et al. 2003; Tanaka et al. 2005). The elevated expression of gene encoding AGPase enzyme in tuberous roots of *M. esculenta* has been shown to enhance biomass yield (Ihemere et al. 2006). The fundamental genetic mechanism controlling their formation in *Rehmannia glutinosa* has been studied to decipher the role of tuberous root development genes (Sun et al. 2010). With the advent of new technologies, a comprehensive analysis of transcriptome and proteome datasets in *R. glutinosa* provided new insight on the mechanisms for the formation of storage organ (Li et al. 2015). However, the knowledge on developmental pattern of tuberous roots in *A. heterophyllum* is limited. The development of tuberous roots in this plant species undergoes simple, yet unique process. After seed germination, the primary roots grow immediately from the radicle and transform directly into tuberous roots rather formed into adventitious roots as in other plant species (Pal et al. 2015). Therefore, the tuberous root formation in *A. heterophyllum* provides a unique system to explore mechanism of sink tissue formation and development vis-à-vis accumulation of root biomass.

The tuberous roots of *A. heterophyllum* contain primary metabolites including starch and other carbohydrates besides aconites, the secondary metabolites found in the buttercup family (Ukani et al. 1996; Rana et al. 2010). Starch biosynthesis genes like AGPase and β-amylase have shown changes in expression pattern during various tuberous root developmental stages in *I. batatas* (Wang et al. 2006). Carbohydrate metabolism pathways were found to be activated in storage roots of *Raphanus sativus* (Mitsui et al. 2015). Next-generation sequencing (NGS) transcriptomes analysis of *A. heterophyllum* root and shoot tissues identified plausible candidate genes responsible for tuberous root formation and development through in silico expression profiling (Pal et al. 2015), which have been further assessed for their association with growth and developmental mechanism of tuberous roots. Fragments per kilobase of transcript per million mapped fragment (FPKM) based expression profiling study was done to identify differentially regulated genes involved in root biomass enhancement. Genes pertaining to primary metabolism in plants, such as starch production, photosynthesis, hormone metabolism and transcription factors were also studied to associate their role in storage organ development and root biomass production in *A. heterophyllum*.

## Materials and methods

### Plant material

Seeds of *A. heterophyllum* were germinated in the nursery of Himalayan Forest Research Institute at Shilaru, Himachal Pradesh, India (2450 m altitude, 31°23′N, 77°44′E) under natural conditions. As the plant starts developing tuberous roots immediately after seed germination, roots of different age groups (6–36 months old) comprising young, intermediate and mature stages were harvested at same time. These were classified as R1, R2, R3, R4 and R5 for 6, 12, 18, 24 and 36 months old tuberous roots, respectively (Pal et al. 2015). Plants of *A. heterophyllum* were also procured for collection of roots and shoots separately for gene expression analysis between both tissues. All samples were frozen immediately in liquid nitrogen and stored at −80 °C until use.

### Transcriptome mining for tuberous root development genes

Assembled NGS transcriptomes of *A. heterophyllum* were downloaded from the http://14.139.240.55/NGS/download.php. The transcriptome data sets corresponding to root and shoot tissues were analyzed using in-house developed scripts along with other bioinformatics approaches. The overall picture for defining parameters of tuberous root formation and development is still not clear in *A. heterophyllum*. Therefore, on the basis of literature survey (Table 1), genes belonging to various physicochemical processes required for tuberous root growth and biomass enhancement were mined in the transcriptomes of *A. heterophyllum* on the basis of high sequence similarity with known genes (≥40 % and E-value threshold of 1e−5). Total of 18 genes, including nine genes belonging to starch pathway (AGPase), photosynthesis (PEP C, POP, PC, RCA), hormone metabolism (HOG1, ARF2) and transcription factor (NAC1, ANT) families were selected in addition to nine genes from our previous study (Pal et al. 2015) for investigating their role in sink tissue development for biomass enhancement. Fragments per kilobase of transcript per million mapped fragment (FPKM) approach was used to calculate the transcript abundance of each gene (Pal et al. 2015, Supplementary Table 1).

**Table 1:** Genes implicated in biomass productivity in different plant species

Category	Sub-category	Genes	Plant species	Source
Tuberous root formation		GMPase SHAGGY NOP10 Expansin Early Nod RBX1 MAP kinase SRF	*Ipomoea batatas* *Manihot esculenta* *Rehmannia glutinosa*	Conklin et al. (1999), Li et al. (2001, 2012), Tanaka et al. (2005), Wang et al. (2005), Dreher and Callis (2007), Sun et al. (2010)
Primary metabolism	Starch pathway	AGPase β-Amylase	*M. esculenta* *I. batatas*	Ihemere et al. (2006), Wang et al. (2006)
	Photosynthesis	PEP C POP PC RCA	*Oryza sativa* *Solanum tuberosum*	Matsuoka et al. (2001), Häusler et al. (2001)
	Hormonal regulation	HOG1 ARF2	*Arabidopsis thaliana*	Okushima et al. (2005), Godge et al. (2008)
	Transcription factors	NAC1 ANT	*A. thaliana*	Xie et al. (2000)

### Genomic DNA and total RNA isolation

Genomic DNA was isolated from leaf tissue of *A. heterophyllum* following the protocol of Murray and Thompson (1980). Total RNA from all samples was isolated using TRIzol^®^ Reagent (Life Technologies, USA) followed by RNase free DNase treatment (Takara Bio Inc, China) according to the manufacturer’s instructions. RNA integrity was assessed in 1 % (w/v) ethidium bromide-stained agarose gel and concentration was estimated using a Nanodrop 2000 spectrophotometer (Thermo Scientific, USA).

### Quantitative real-time PCR (qPCR) analysis

Primer pairs for 18 genes were designed using Primer 3 (http://bioinfo.ut.ee/primer3-0.4.0/) (Supplementary Table 2) and tested on genomic DNA and cDNA of *A. heterophyllum*. cDNA synthesis was done using Verso cDNA synthesis kit (Thermo Scientific, USA) from total RNA (5 µg) as per manufacturer’s instructions. The cDNAs were then separated by electrophoresis, stained with ethidium bromide to further verify equal concentrations (100 ng each). The reaction was performed in triplicate on a CFX96 system (Bio-Rad Laboratories, Hercules, CA, USA) with the iScript one step RT PCR kit (Bio-Rad). The PCR protocol was as follows: denaturation for 5 min at 94 °C, followed by 40 cycles each of denaturation for 20 s at 94 °C, annealing for 30 s at 48–52 °C, followed by one elongation step for 20 s at 72 °C. 26S rRNA and GAPDH were used as internal controls for calculating transcript abundance. The significant differences between replicates were statistically evaluated by standard deviation in the form of error bars as mean ± SD for data recorded in triplicates (repeated thrice).

### Statistical analysis

Cluster analysis was done for gene expression profiles obtained from qPCR data with the help of *K*-means clustering, Agglomerative hierarchical clustering (AHC) and Gaussian mixture model (GMM) methods (Yu and Qin 2008; Jain 2010). Its aim was to establish correlation between abundance of the transcripts and the corresponding tuberous root developmental stages. *K*-means clustering aims to partition *N* observations into *K* clusters in which each observation belongs to the cluster with the nearest mean, serving as a prototype of the cluster. AHC generates dendrogram which represents a hierarchy of partitions. It is then possible to choose a partition by truncating the tree at a given level, the level depending upon user-defined constraints. Fitted GMMs cluster by assigning query data points to the multivariate normal components that maximize the component posterior probability of the given data set.

## Results and discussion

### Expression analysis of tuberous root development genes

**Fig. 1:**
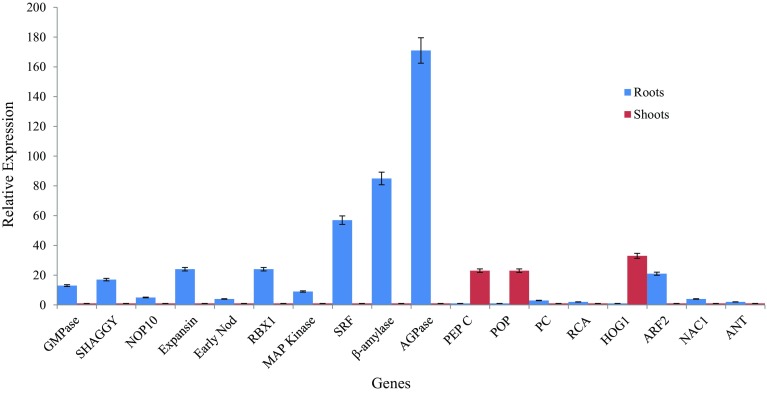
Expression status of tuberous root development genes in different tissues (roots and shoots) of *A. heterophyllum*. *Error bars* represent mean ± SD for data recorded in triplicates (repeated thrice)

To gain an insight into the molecular basis of sink organ, i.e., tuberous root development in *A. heterophyllum*, quantitative expression of 18 genes was studied in different tissues using transcript abundance analysis. qPCR analysis revealed that 15 out of 18 genes of aforementioned classes had relatively higher expression in roots of *A. heterophyllum* compared to shoots. Eight genes encoding GMPase, SHAGGY, Expansin, RBX1, SRF, β-amylase, AGPase and ARF2 showed elevated level of transcripts in roots (13–171 folds) compared to shoots (Fig. 1). The transcripts of AGPase, β-amylase and SRF genes showed highest transcript abundance of 171-, 85- and 57-fold, respectively, in roots. Relatively higher expression of genes coding for AGPase and β-amylase enzymes was positively correlated to their role in starch biosynthesis in *M. esculenta* (Ihemere et al. 2006; Saithong et al. 2013), *I. batatas* (Wang et al. 2005) and model crop species like *Oryza sativa* and *Zea mays* (Geigenberger 2011). Additionally, the starch production is regulated by the activity of AGPase, the first enzyme of the starch biosynthetic pathway (Li et al. 2012). The transcript of SRF gene increased 57-fold in roots compared to shoots. The gene has been found to developmentally regulate storage root formation in *I. batatas* by actively initiating cell division process, thereby causing thickening of tuberous roots (Tanaka et al. 2005). The higher expression of this gene has been shown to regulate plant carbohydrate metabolism in *I. batatas* (Li and Zhang 2003). The genes encoding for RBX1 and Expansin enzymes showed 24-fold transcript increase in the roots of *A. heterophyllum*. The ubiquitination activities, which are primarily active during tuberous root development, are controlled by RBX1 enzyme (Dreher and Callis 2007). Similarly, Expansin is well known regulator of cell wall extension during sink organ development in *R. glutinosa* (Sun et al. 2010). The results of this analysis were in agreement with the in silico expression profiles of nine genes reported in Pal et al. (2015).

Furthermore, three genes encoding PEP C, POP and HOG1 showed highest transcript abundance of 23-, 23- and 33-fold, respectively, in shoots of *A. heterophyllum* compared to roots which was evident as far as whole plant development is concerned. These results were comparable to the role of major pathways of primary metabolism towards plant growth and development. For example, photosynthesis genes, PEP C and POP have been shown to influence photosynthetic capacity and seed yield in *O. sativa* (Matsuoka et al. 2001). Moreover, over expression of these genes regulated cytosolic enzymatic levels of photosynthetic pathway resulting in yield enhancement of *Solanum tuberosum* (Häusler et al. 2001). Cytokinin production in *Arabidopsis thaliana* has been found to be regulated by HOG1 gene which significantly control biomass yield (Godge et al. 2008).

**Fig. 2:**
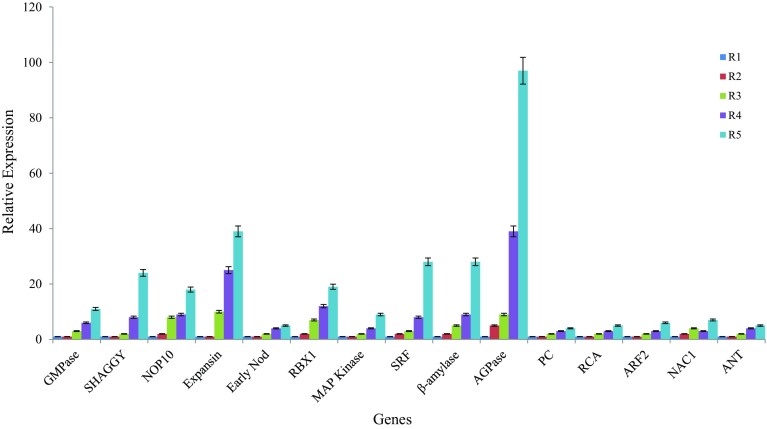
Expression status of tuberous root development genes in tuberous root developmental stages (R1–R5) of *A. heterophyllum*. *Error bars* represent mean ± SD for data recorded in triplicates (repeated thrice)

The relative role of 15 genes showing higher expression in roots was further validated on different developmental stages of tuberous root formation in *A. heterophyllum*. This was done to ascertain their significance in root biomass increase by screening all stages, from young rootlet to fully developed tuberous roots. Comparative expression analysis of tuberous root development stages (see “Materials and methods”) revealed that almost all genes showed increase in transcript abundance in R4 and R5 stages compared to R1, R2 and R3 stages. The results showed nonsignificant changes in the expression levels of eight genes coding for GMPase, SHAGGY, NOP10, Expansin, RBX1, AGPase, β-amylase and SRF enzymes from stages R1–R3, significant increase in intermediate R4 stage (6–39 folds) and dramatic increase in their transcript levels in fully developed mature tuberous roots of R5 stage (11–97 folds) in *A. heterophyllum* (Fig. 2). The precise reasons for variations in transcript abundance of these genes are not clear; however, the capacity of sink organ to biosynthesize and accumulate primary metabolites have been known to increase with age of the plant. For example, carbohydrate metabolism and starch accumulation were found to increase with tuberous root development in *R. sativus* (Mitsui et al. 2015) and *I. batatas* (Wang et al. 2015), respectively. The transcript of AGPase gene showed 97-fold expression in R5 stage compared to R1 stage. This could be related to the formation of fully developed sink tissue with increased storage ability for the accumulation of starch and other sugars (Saithong et al. 2013). Being a rate-limiting enzyme of starch biosynthesis, the expression pattern of AGPase gene was consistent with the published reports (Yu et al. 2010). Similarly, the 28-fold increase in transcript abundance of gene coding for β-amylase enzyme corresponds to its regulatory role in starch biosynthetic pathway (Buléon et al. 1998). The expression of SRF gene showed 28-fold increase in R5 stage compared to R1 stage. Previous reports have suggested that the mature storage roots of *I. batatas* (Tanaka et al. 2005) accumulate carotenoids, therefore, it was observed that the expression of SRF gene increases with the formation of fully developed tuberous roots having utmost rate of primary metabolism. Interestingly, the change in expression pattern of Expansin transcript was indeed an unforeseen finding. It showed 39-fold increase in R5 stage than R1 stage while its expression remained 24-fold in roots compared to shoots. The increase in its transcript level is attributed to its involvement in various biochemical and physiological processes for tuberous root development including root hair formation for rapid cell proliferation in various plant species (Huang et al. 2001; Li et al. 2003). The transcripts coding for SHAGGY and NOP10 genes showed 24- and 18-fold increase in expression level in R5 stage compared to R1 stage. SHAGGY has been known to be involved in plant developmental processes (Li et al. 2001) while NOP10 regulates mRNA splicing and ribosome biogenesis (Meier 2005), but its exact role in plant processes remains to be elucidated. Further, NAC1 and ANT are known for the biosynthesis and accumulation of primary metabolites towards biomass production in plants, but surprisingly, their transcripts did not showed significant increase in expression with increase in the root biomass. This could be attributed to their role in regulating cell proliferation and organ growth by maintaining the meristematic competence of organ cells (Xie et al. 2000; Hu et al. 2003), although molecular mechanism behind such developmental signals is largely undefined and can be fully ascertained through gene function approaches.

**Fig. 3:**
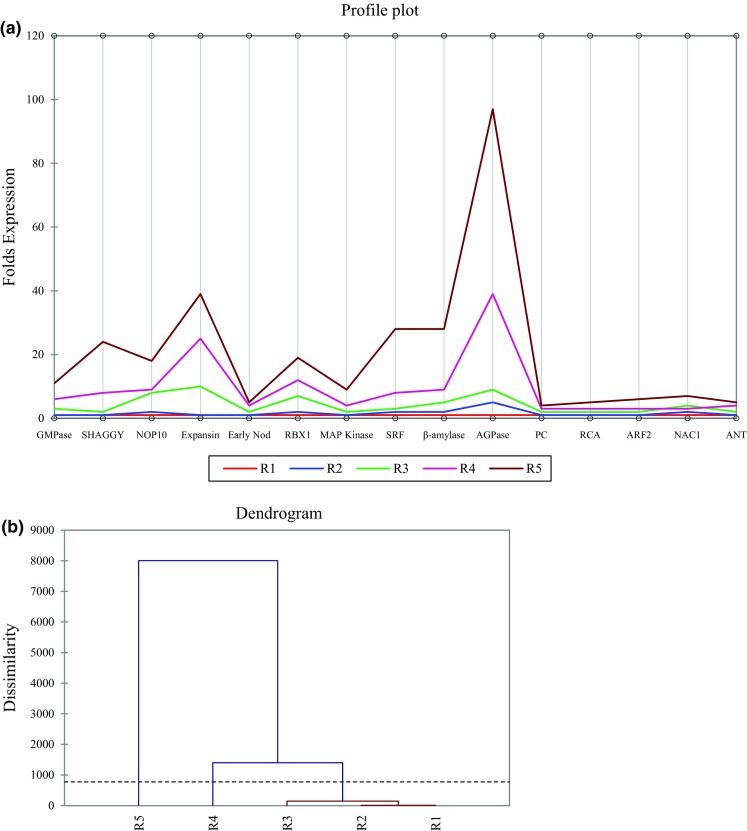
Cluster analysis of tuberous root development-related genes in *A. heterophyllum* through **a** *K*-means clustering, **b** agglomerative hierarchical clustering. K-means clustering indicated increased profiles for R4 and R5 stages while AHC reflected close proximity between R1–R3 stages using dissimilarities. *Broken line* in AHC marks the level of truncation during clustering

For complete corroboration of the current findings, the data set was subjected to statistical analysis. The study showed positive correlation with the gene expression data for different stages (R1–R5) of tuberous root development in *A. heterophyllum*. Clustering of the whole data set revealed close relationship between R1, R2 and R3 stages showing more or less similar expression pattern while R4 and R5 stages exhibited increase in transcript abundance as plant attained maturity. The observed profiles of eight genes viz. GMPase, SHAGGY, NOP10, Expansin, RBX1, SRF, β-amylase and AGPase were found to be distinct and well separated from each other in R5 stage compared to R4 stage. The results obtained by *K*-means clustering and agglomerative hierarchical clustering (AHC) demonstrate this scenario very well (Fig. 3). Profile plot through *K*-means clustering indicated increased profiles for R4 and R5 stages while bottom up approach for clustering in AHC clearly reflected the close proximity of R1–R3 stages. Additionally, the Gaussian mixture models (GMM) for eight genes with high expression levels have ascertained their importance in the formation of tuberous roots (Supplementary Fig. 1). Overall, multiple genes of different families exhibited an important role in tuberous root architecture for biosynthesis and storage of primary metabolites, thus paving the way towards increasing the biomass yield through genetic modification in *A. heterophyllum*.

In conclusion, the results obtained from this study demonstrated the apparent role of tuberous root development genes for the biomass production in *A. heterophyllum*. There is a possibility of altering the expression levels of key genes by gene function approaches for improving tuberous root (biomass) yield for herbal drug industries. These results can be further explored to dissect the molecular regulation of tuberous root formation and growth in *A. heterophyllum*.

## Electronic supplementary material

Below is the link to the electronic supplementary material.

Supplementary material 1 (DOCX 14 kb)

Supplementary material 2 (DOCX 14 kb)

Supplementary material 3 (DOCX 1130 kb)

## Abbreviations

AGPase: ADP-glucose pyrophosphorylase
ARF2: Auxin responsive factor 2
GMPase: GDP-mannose pyrophosphorylase
HOG1: HOG1 protein kinase 1
MAP K: MAP kinase
NOP10: H/ACA ribonucleoprotein complex
PC: Plastocyanin
PEP C: Phosphoenolpyruvate carboxylase
POP: Pyruvate orthophosphate
RBX1: RING-box protein 1
RCA: RuBisCO activase
SRF: SRF receptor kinase
