# Supplementary material for: Transcriptome-wide mining suggests conglomerate of genes associated with tuberous root growth and development in Aconitum heterophyllum Wall
Source: 3 Biotech. 2016 Jul 11;6(2):152. doi: 10.1007/s13205-016-0466-y (PMC4940232; doi:10.1007/s13205-016-0466-y)
Supplement: Supplementary file 1 — Supplementary material 1 (DOCX 14 kb) [file 13205_2016_466_MOESM1_ESM.docx]

**Supplementary Table 1** *In silico* **t**ranscript abundance of primary metabolism genes in transcriptomes of *A. heterophyllum*.

| Gene | Accession Number | Transcript ID | Length | Effective Length | Expected Count | TPM | FPKM |
| --- | --- | --- | --- | --- | --- | --- | --- |
| AGPase | AY184483.1 | Transcript_9990 | 1905 | 1789.35 | 12658.91 | 137.75 | 180.56 |
| PEP C | X55664.1 | Transcript_5167 | 2399 | 2199.35 | 1958.23 | 39.11 | 49.67 |
| POP | NM_001112268.2 | Transcript_5527 | 3219 | 3152.35 | 1989.34 | 40.06 | 49.92 |
| PC | M20937.1 | Transcript_29225 | 693 | 504.35 | 344.79 | 7.86 | 9.04 |
| RCA | AM748404.1 | Transcript_13197 | 1198 | 965.35 | 436.25 | 8.65 | 9.86 |
| HOG1 | AY885075.1 | Transcript_38622 | 502 | 410.35 | 1106.44 | 28.36 | 33.72 |
| ARF2 | AF336918.1 | Transcript_29911 | 366 | 288.35 | 111 | 2.67 | 3.13 |
| NAC1 | KJ572407.1 | Transcript_32041 | 588 | 462.36 | 234.58 | 4.12 | 4.99 |
| ANT | U40256.1 | Transcript_68747 | 981 | 799.36 | 400.06 | 7.23 | 9.34 |
