# Supplementary material for: Transcriptome-wide mining suggests conglomerate of genes associated with tuberous root growth and development in Aconitum heterophyllum Wall
Source: 3 Biotech. 2016 Jul 11;6(2):152. doi: 10.1007/s13205-016-0466-y (PMC4940232; doi:10.1007/s13205-016-0466-y)
Supplement: Supplementary file 2 — Supplementary material 2 (DOCX 14 kb) [file 13205_2016_466_MOESM2_ESM.docx]

| **Gene** | **Forward Primer** | **Reverse Primer** | **Annealing temperature (°C)** | **Fragment size (bp)** |
| --- | --- | --- | --- | --- |
| 26S rRNA | 5′-CACAATGATAGGAAGAGCCGAC-3′ | 5′-CAAGGGAACGGGCTTGGCAGAATC-3′ | 58 | 500 |
| GAPDH | 5′-TTGCCATCAATGACCCCTTCA-3′ | 5′-CGCCCCACTTGATTTTGGA-3′ | 56 | 215 |
| GMPase | 5′-TTGAGGCCTTTGACACTCAG-3′ | 5′-ATCTTGATCCCAACCTTTGC-3′ | 50 | 140 |
| SHAGGY | 5′-ACGACCAAGTGGATCAACAA-3′ | 5′-TCTGCCCACTTGAGATCTTG-3′ | 49 | 170 |
| NOP10 | 5′-AGCAAGGAGAGGAGAAACCA-3′ | 5′-AGATGAGCACAACTGCCAAG-3′ | 50 | 170 |
| Expansin | 5′-ACTCCCACCTCCACTAATGC-3′ | 5′-TTCTAGTCTGGAGCCCAGGT-3′ | 50 | 120 |
| Early Nod | 5′-TACATTGTCGCTCGAGGAAG-3′ | 5′-AAAGCGACCAAGCGAAGTAT-3′ | 48 | 100 |
| RBX1 | 5′-CTGCAATTGAATCCCCTTTT-3′ | 5′-TTTTCCGTTGCTCAGTCAAG-3′ | 51 | 110 |
| MAP K | 5′-TACCACGATCCCTCTGATGA-3′ | 5′-TCTTGTTCGTTCGCTTTGTC-3′ | 50 | 100 |
| SRF | 5′-CCTAAGCCCAACGACAATTT-3′ | 5′-AGGCTTCAGAAGGAGGTTGA-3′ | 50 | 100 |
| β-amylase | 5′-CTGAGTGCAGGTTGGAGAGA-3′ | 5′-TTGGTGGTCCGTCTTTGTTA-3′ | 50 | 140 |
| AGPase | 5′-CAATTGGTGGATGCTACAGG-3′ | 5′-TATATGTGCGTGCAATGTGG-3′ | 50 | 150 |
| PEP C | 5′-GCCATCGAGATGATGTATGC-3′ | 5′-TGGTCCTGATCTTCCAACAA-3′ | 49 | 150 |
| POP | 5′-TGAGCACCGTTGTTTCTAGC-3′ | 5′-GGGTCAACAGGAGAAGTGGT-3′ | 48 | 110 |
| PC | 5′-TCCCAACAACTTTACGGTGA-3′ | 5′-TAGAAATCTTGGCAGCATCG-3′ | 52 | 110 |
| RCA | 5′-GTGGGGTTTGGAAGAAGAAA-3′ | 5′-TTGCAAATGCTACCAACCAT-3′ | 50 | 110 |
| HOG1 | 5′-AGACCCTGCAGGAGTACTGG-3′ | 5′-TGGATCAAAAGCGTAGCATC-3′ | 50 | 110 |
| ARF2 | 5′-TCCACCCAGTTGTAAGCAAA-3′ | 5′-GAATCCACCTTGGCAAGAAT-3′ | 50 | 110 |
| NAC1 | 5′-TTCACCACCCTCAAGAACAA-3′ | 5′-ACCCTAAGAAAGGAGCAGCA-3′ | 52 | 170 |
| ANT | 5′-CCTTCACCCATCCTCAGATT-3′ | 5′-CCAGTAGTGGAGGTGGAGGT-3′ | 50 | 120 |

**Supplementary Table 2** Primer sequences used in qPCR analysis of tuberous root development genes.
