# Supplementary material for: Transcriptome-wide mining suggests conglomerate of genes associated with tuberous root growth and development in Aconitum heterophyllum Wall
Source: 3 Biotech. 2016 Jul 11;6(2):152. doi: 10.1007/s13205-016-0466-y (PMC4940232; doi:10.1007/s13205-016-0466-y)
Supplement: Supplementary file 3 — Supplementary material 3 (DOCX 1130 kb) [file 13205_2016_466_MOESM3_ESM.docx]

**Supplementary Fig. 1** Gaussian mixture models (GMM) for 8 genes with high expression levels for tuberous root development in *A. heterophyllum*. Density estimation of 8 genes was carried out by optimizing expectation maximization (2-component BIC criterion).
